# Frontal Lobe Contusion in Mice Chronically Impairs Prefrontal-Dependent Behavior

**DOI:** 10.1371/journal.pone.0151418

**Published:** 2016-03-10

**Authors:** Austin Chou, Josh M. Morganti, Susanna Rosi

**Affiliations:** 1 Brain and Spinal Injury Center, University of California, San Francisco, CA, United States of America; 2 Neuroscience Graduate Program, University of California, San Francisco, CA, United States of America; 3 Department of Physical Therapy Rehabilitation Science, University of California, San Francisco, CA, United States of America; 4 Department of Neurological Surgery, University of California, San Francisco, CA, United States of America; Rutgers University, UNITED STATES

## Abstract

Traumatic brain injury (TBI) is a major cause of chronic disability in the world. Moderate to severe TBI often results in damage to the frontal lobe region and leads to cognitive, emotional, and social behavioral sequelae that negatively affect quality of life. More specifically, TBI patients often develop persistent deficits in social behavior, anxiety, and executive functions such as attention, mental flexibility, and task switching. These deficits are intrinsically associated with prefrontal cortex (PFC) functionality. Currently, there is a lack of analogous, behaviorally characterized TBI models for investigating frontal lobe injuries despite the prevalence of focal contusions to the frontal lobe in TBI patients. We used the controlled cortical impact (CCI) model in mice to generate a frontal lobe contusion and studied behavioral changes associated with PFC function. We found that unilateral frontal lobe contusion in mice produced long-term impairments to social recognition and reversal learning while having only a minor effect on anxiety and completely sparing rule shifting and hippocampal-dependent behavior.

## Introduction

Traumatic Brain Injury (TBI) is a leading cause of long-term neurological disability in the world with over 3 million TBI-related emergency and outpatient visits a year in the United States alone [1, 2]. While the fatality rate after TBIs has declined in the last two decades, the incidence of TBIs continues to climb, and the majority of TBI patients experience prolonged neurocognitive dysfunctions that substantially impact their quality of life [3, 4]. Clinically, most TBIs are mild to moderate in severity, result in damage to frontal lobe areas due to cortical contusion, and affect prefrontal cortex (PFC)-dependent functions [5–7].

The PFC is responsible for executive function and is most susceptible to injury [5, 8]. Deficits in executive function in TBI patients include poor sociability [9], loss of cognitive flexibility [10, 11] and increased anxiety [12, 13]. The vulnerability of the PFC to TBI is further highlighted by studies correlating behavioral impairment to the severity of TBI characterized by MRI scans [5, 14]. Yet despite the relevance of PFC function in TBI patients, limited attention has been devoted to understanding and modeling the damage processes in the PFC at a chronic time point after a TBI event.

To more closely recapitulate injuries observed in human patients, we applied the controlled cortical impact (CCI) method to induce a frontal cortex TBI and measured PFC function at chronic time points post injury. Using the three-chamber sociability task [15, 16], the rule shift paradigm [17], and the elevated plus maze [18], we determined the injury’s effect on social behavior, cognitive flexibility, and anxiety respectively. Furthermore, we utilized the novel object recognition task [19] to determine whether frontal lobe injury would affect hippocampal-dependent function given the anatomical and functional link between the two regions [20]. Our results demonstrate that frontal lobe contusion impairs social recognition and orbitofrontal cortex (OFC)-dependent rule reversal behavior. Conversely, the model displays only a trend for increased anxiety and no impairment on medial prefrontal cortex (mPFC)-dependent rule shifting or hippocampal-dependent memory.

## Materials and Methods

### Animals

All experiments were conducted in accordance with National Institutes of Health *Guide for the Care and Use of Laboratory Animals* and were approved by the Institutional Animal Care and Use Committee of University of California (San Francisco). All C57B6/J wildtype (WT) male mice were purchased from Jackson Laboratory (Bar Harbor, ME) and used for experiments at approximately 12 weeks of age. Mice were group housed in environmentally controlled conditions with reverse light cycle (12:12h light:dark cycle at 21 ± 1°C) and provided food and water *ad libitum*.

### Surgical Procedure

All animals were randomly assigned to TBI or sham surgeries. Animals were anesthetized and maintained at 2% isoflurane and secured to a stereotaxic frame with non-traumatic ear bars. The hair on their scalp was removed, and eye ointment and betadine were applied to their eyes and scalp respectively. A midline incision was made to expose the skull. TBI was induced in the frontal lobe using the controlled cortical impact (CCI) model [21]. Mice received a craniectomy ~2.5 mm in diameter using an electric microdrill. The coordinates of the craniectomy were: anteroposterior, +2.34 mm and mediolateral, +1.62 mm with respect to bregma. Any animal that experienced excessive bleeding due to disruption of the dura was removed from the study. After the craniectomy, the contusion was induced using a 2 mm convex tip attached to an electromagnetic impactor (Leica). The contusion depth was set to 1.25 mm from dura with a velocity of 4.0 m/s sustained for 300 ms. These injury parameters were chosen to target, but not penetrate, the medial and orbitofrontal regions of the PFC. After injury, the scalp was sutured and the animal was allowed to recover in an incubation chamber set to 37°C. Animals were returned to their home cage after showing normal walking and grooming behavior. Sham animals received craniectomy surgeries but without the CCI injury. On average animals recovered and were ambulatory within 10 minutes of removal from isoflurane exposure. After surgery animals were weighed weekly and monitored for indications of poor health including ambulation, muscle atrophy and emaciation, lethargy, infection of surgery site, anorexia, difficulty breathing, and difficulty remaining upright. Any animals that displayed such signs were monitored closely, treated if possible, and humanely euthanized based on veterinarian recommendation. Animals fully recovered from the surgical procedures as exhibited by normal behavior and weight maintenance.

### Behavioral Assays

#### Social Approach Task

At 29 d after surgery, a group of animals(n = 16-17/group) were tested for sociability and social recognition behavior on the three-chamber social approach task as previously described [22]. Animals were placed individually into the center of a three-chamber environment and their behavior was recorded during three consecutive phases: habituation, sociability, and social recognition. During habituation phase, mice were allowed to explore the entirety of the empty environment for 10 minutes. Following habituation, mice were guided to the center chamber, and access to the chambers was blocked.

During the sociability phase, animals were tested for their preference for a novel mouse over a novel object. A stranger mouse from a different cage was placed into a small cylindrical cage on either the left or right chamber while a similar, but empty, cage was placed in the opposite chamber. Placement of stranger mouse into the right or left cage, was chosen semi-randomly for each trial. Test mice were allowed to explore the stranger mouse and an empty cage for 10 minutes before being restricted in the center chamber again. During the social recognition phase, animals were tested for their preference for a novel mouse over a familiar mouse they previously encountered. In this phase, a new stranger animal from a third cage was placed into the previously empty cage. Test mice were allowed 5 minutes to explore the environment again, this time with the familiar mouse (from the sociability phase) and the new stranger mouse. The environment and cage objects were cleaned with 0.025% bleach between test animals. Exploration was recorded using the previously mentioned video tracking and analysis setup (Ethovision XT 8.5, Noldus Information Technology) and analyzed for time spent interacting with the stimuli during the first three minutes of each phase. The Preference Ratio was calculated as the percent of time exploring the mouse vs percent of time exploring the object (empty cage) for phase 2 and as percent of time exploring the novel mouse vs percent of time exploring the familiar mouse for phase 3.

#### Rule Shift Assay

At 35–40 days post injury, animals (n = 7/group) were tested for cognitive flexibility using the rule shift assay based upon the set shift paradigm as described by Bissonette et al. (2008) and the rule-shifting assay as described by Cho et al. (2015). Mice were housed either individually or in pairs and placed under food restriction for two days such that mice reached 80–85% of their starting body weights. During food restriction, mice were exposed to all assay components (e.g. bowls, textures, odors). The provided food during the assay consisted of normal chow and the food reward–pieces of Honey Nut Cheerios (General Mills). Both were placed in the bowls to associate the bowls with food and covered with prepared digging media.

The bowls used for the assay were purple, small Lixit Nibble Food Bowls (Amazon). The digging media consisted of two dimensions: odor and texture. The olfactory cues consisted of dried, ground spices: garlic powder, onion powder, paprika, and coriander (Simply Organic). Unscented digging media were obtained from local hardware and pet stores and the animal facility (Mosser Lee decorative white sand, unbranded unscented cat litter, alpha-dry bedding, or hardwood). The digging media and odors were utilized in two different combinations (e.g. Combination 1: sand and litter with garlic and coriander. Combination 2: alpha-dry bedding and hardwood with paprika and coriander). Pairing of digging media and odors were made with 0.7% odor and 0.1% ground Cheerios by volume.

During habituation and test trials, mice were placed into a holding cage while the home cage was used as the test chamber [17]. In the test chamber, mice were presented with two bowls, each filled with a different texture-odor pair from a single combination. Only one of the bowls contained the food reward. During habituation, mice were given 10 consecutive trials. Mice were allowed three minutes to dig in both bowls to learn that only one bowl contained food on each trial. The animals were considered to have timed out of a trial if they failed to dig within the three minutes. Any animals that timed out of five trials on a single day were excluded from the study. The animals were exposed to every pair from both media combinations, and the food reward and cues had no correlation during habituation to prevent animals from forming any associations prior to the test days.

During test days, the mice were exposed to only a single media combination. They were allowed three minutes to explore the two bowls until they began digging, signifying a choice. If they correctly chose the rewarded bowl, they were returned to the holding cage after they had found and consumed the food reward. If they chose the incorrect bowl, they were returned to the holding cage after they had given up digging in the selected bowl without a chance to dig in the bowl with the reward and the trial was marked as an error. If they did not dig within the three minutes, they were returned to the holding cage and the trial was marked as a timed out trial.

Animals first had to learn an initial association where the food reward was associated with one of the four cues presented. The learning criterion was defined as eight successful choices in the last ten consecutive attempts. After the animal had learned the initial association, the rule was either reversed (e.g. texture to texture) or shifted (e.g. texture to odor). The animals continued with the trials until they met the learning criterion again for the new association. The reward was presented equally on the left and right of the test chamber and the order of the possible media pairs were pseudo-randomized through the assay such that animals were exposed to a media pair up to a maximum of three consecutive trials. The animals did not exhibit a preference for any cue over the others before forming initial associations (data not shown).

Animals were given three test days. On day 1, media combination 1 was used with a rule reversal after Initial Association. On day 2, media combination 2 was used with a rule reversal. On day 3, media combination 2 was used again but with a rule shift. The total errors to reach criterion were analyzed.

Animals were divided into two groups and tested on the rule shift assay either 35–40 days (n = 7/group) or 5.5 months post-injury (n = 10-11/group).

#### Elevated Plus Maze

At 28 d after surgery, animals were tested for anxiety on the elevated plus maze (EPM; n = 21–22). The EPM consists of two exposed, open arms (35cm) opposite each other and two enclosed arms (30.5cm) also across from each other. The four arms are attached to a center platform (4.5 cm square) and the entire maze elevated 40cm off the floor [23]. Mice were placed individually onto the center of the maze and allowed to explore the maze for 5 minutes. Their activity was recorded using an overhead camera connected to a video tracking and analysis system (Ethovision XT 8.5, Noldus Information Technology). The maze was cleaned with 0.025% bleach between animals.

#### Novel Object Recognition

At 31 d after surgery, a separate cohort of animals (n = 9) from those that ran the previous assays were tested for hippocampal-dependent memory function using a mouse novel object recognition assay [19]. The test environment consists of an open field arena in a dimly lit behavioral testing room. Mice were allowed to explore the arena for two 10 minute periods for two consecutive days (habituation phase). On day three (training phase), two identical objects (red lego blocks) were secured to the floor in opposite corners of the arena using magnets and mice were allowed to explore the arena and objects for 5 minutes. 24hours later on day four (testing phase), one of the objects was replaced with a novel object (orange lego flower) of similar dimensions and texture. Mice were reintroduced in to the arena and allowed to explore for 5 minutes. The objects and arena were cleaned with 0.025% bleach between trials and animals. Trials were recorded using the video tracking and analysis setup (Ethovision XT 8.5, Noldus Information Technology) and manually scored. Exploratory behavior was defined as time the animals spent directing its nose towards an object within 3 cm of the object. Data was expressed as percent of time mice spent exploring each object. Mice that had less than 5 seconds of exploration time during either training or testing were excluded from analysis.

### Tissue Collection

All mice were lethally overdosed using a mixture of ketamine (150mg/kg) and xylaxine (15mg/kg). Once animals were completely anesthetized, the chest cavity was opened and each animal was transcardially perfused with ice-cold Hank’s balanced salt without calcium and magnesium (HBSS; Gibco) followed by 4% paraformaldehyde (PFA) in buffered saline. Immediately after perfusion, brains were post-fixed in 4% PFA before being transferred to 30% sucrose and stored at 4°C.

### Brain Tissue Sectioning and Imaging

All brain tissues used for imaging was sectioned as previously described (Morganti 2014). 40 μm free-floating sections were stained for neuronal nuclei (NeuN, MAB377, Millipore) with biotinylated secondary antibodies (Biotinylated anti-mouse IgG, BA-2001, Vector) and revealed with DAB (Sigmafast DAB tablets, Sigma-Aldrich). Sections were mounted onto Superfrost Plus slides (Fisher #12-550-15). All imaging was achieved using a Zeiss Imager.Z1 Apotome microscope controlled by ZEN software (Zeiss 2012).

### Lesion Analysis

To calculate cortical volume for each animal, six sections evenly spaced at 240 um apart and centered at the epicenter of impact were analyzed utilizing a Zeiss Imager.M1 Apotome microscope at 2.5x magnification and the Cavalieri estimator in StereoInvestigator 10.0. Grid spacing was set to 200 um with slice cut thickness of 40 um for the Cavalieri estimator. Tissue loss was quantified as the percentage of ipsilateral volume over contralateral volume.

### Data Analysis

Results were analyzed using Prism software (v6.05, GraphPad; La Jolla, CA) and expressed as mean ± standard error of the mean (SEM). Statistical analyses were performed using Student’s t-test with *p* values of <0.05 considered as significant.

## Results

### Frontal CCI produces a protracted contusion in the dorsal frontal cortex

**Fig 1:**
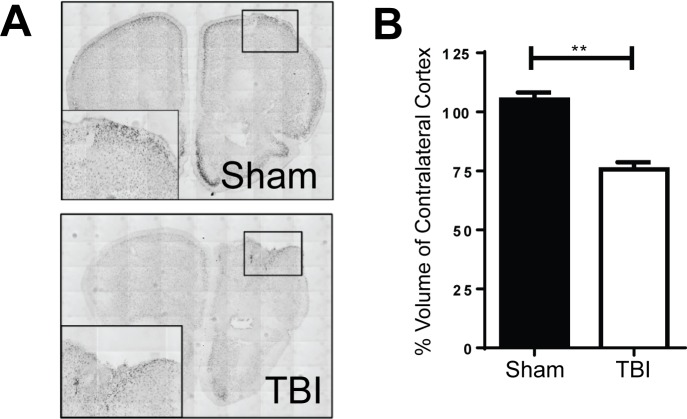
Frontal Lobe CCI results in a cavitation at site of injury. **A.** 40 days post-injury, qualitative NeuN staining reveal normal morphology in sham control animals (top) and a cavitation at site of injury in animals that had received a TBI (bottom). **B.** Injured animals showed approximately 25% cortical tissue loss (n = 3/group; Student’s t-Test; **p < 0.01).

Approximately 40 days after injury and following behavioral assessment, we perfused animals from each treatment group for volumetric analysis. Qualitative images for NeuN staining showed formation of a cavitation at the site of injury (Fig 1A). Quantification through stereological analysis of the frontal lobe revealed approximately 25% loss of cortical volume in injured animals compared to sham (Fig 1B; p<0.01).

### Social recognition but not sociability is impaired by frontal TBI

**Fig 2:**
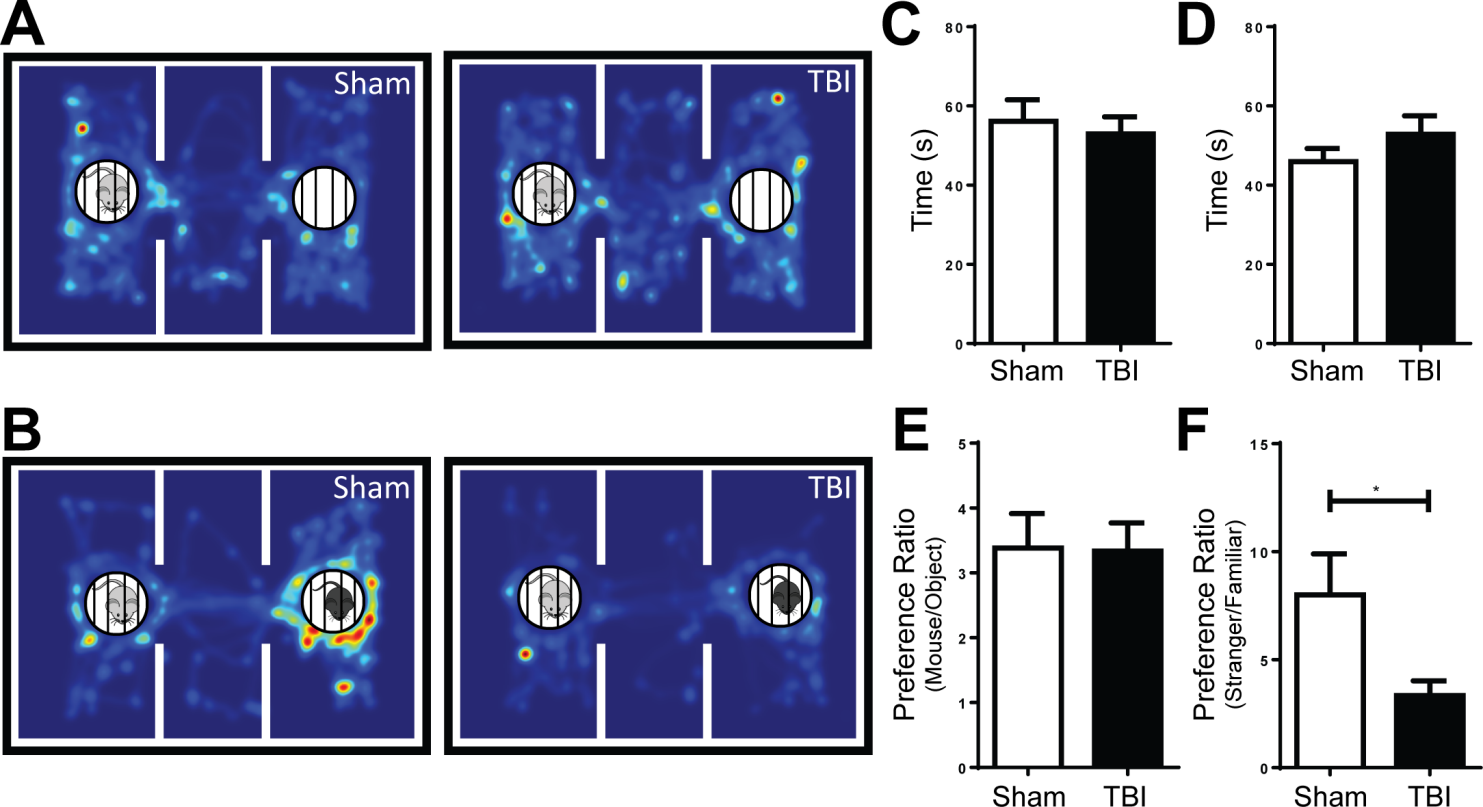
Injured mice demonstrate impairment in social recognition but not sociability on the three-chamber social approach task 1 month post-injury. **A, B.** Top-down heat map of the animal’s nose point location during each phase cumulatively. During sociability (A), animals were exposed to a stranger mouse (left side, light grey) and an empty cage (right chamber). During social recognition (B), animals were exposed to the familiar mouse from the sociability phase (left chamber, light grey) and a new stranger mouse (right chamber, black). **C, D.** There were no differences in time spent exploring the stimuli during sociability (C) or social recognition (D) phases (Student’s t-Test; p > 0.05). **E.** Animals that received a frontal lobe CCI did not exhibit any difference in preference for the mouse over the empty cage (object) during the sociability phase (Preference Ratio>1; Student’s t-Test; p > 0.05). **F.** Animals that received a frontal lobe CCI had significantly less preference for the stranger over the familiar mouse during the social recognition phase (n = 16-17/group; Student’s t-Test; *p < 0.05).

Since social behavior is often altered after TBI events in human patients [14, 24], we utilized the three-chamber social approach task to investigate sociability and social recognition of the mice one month after injury. Mice were tested on two aspects of social behavior: their preference for socializing with a mouse over exploring a novel object (sociability; Fig 2A) and their preference for interacting with a novel mouse over a familiar mouse (social recognition; Fig 2B). There were no differences in total exploration time between treatment groups during either phase (Fig 2C and 2D). Frontal TBI did not affect the sociability of the injured mice; both experimental groups showed strong preferences for interacting with a mouse over an empty cage (Preference Ratio>1; Fig 2A and 2E). Although both groups exhibited preference for the novel stranger over the familiar mouse, however, TBI-injured animals had significantly less preference compared to sham (Fig 2B and 2F; p<0.05).

### Frontal TBI selectively impairs OFC-dependent reversal learning but spares mPFC-dependent rule shift behavior

**Fig 3:**
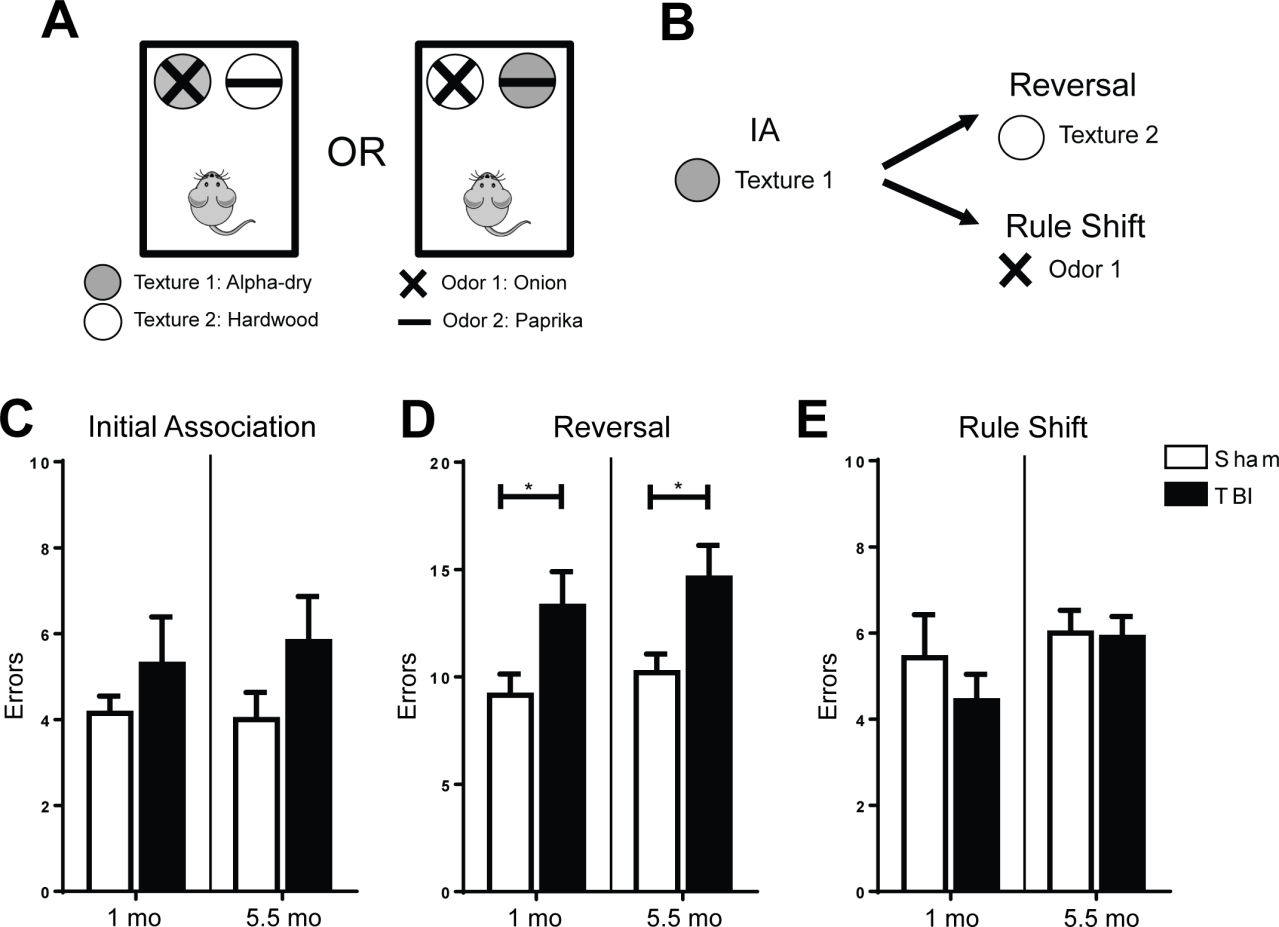
Frontal lobe TBI impairs reversal learning but not rule shifting at 1 month post-injury and the deficit persist at 5.5 months after injury. **A.** Animals were presented with a combination of two textures and two odors during each test day. **B.** Animals first learned to associate a single cue with a food reward. After successfully learning the initial association (IA), animals were tested on either reversal learning or rule shifting which changed the association to a cue within the same or second dimension respectively. **C.** There was no difference between sham and TBI animals on forming an initial association (1mo: n = 7/group; 5.5mo: n = 9-10/group; Student’s t-Test; p > 0.05). **D.** Animals with frontal lobe TBIs made significantly more errors during the reversal task than their sham controls at 1 month after injury. A second cohort exhibited the same effect at 5.5 months after injury (Student’s t-Test; *p < 0.05). **E.** Frontal lobe TBI did not significantly affect rule shifting performance at either 1 month or 5.5 months after injury (Student’s t-Test; p > 0.05).

To determine whether frontal lobe TBI would produce region-specific behavioral deficits, we employed the rule shift assay to identify differences in OFC-mediated or mPFC-mediated behavior after injury [17]. Mice first learned to associate one of four cues (Texture 1, Texture 2, Odor 1, Odor 2) with a food reward and then were tested on their ability to reverse the association (e.g. Texture 1 to Texture 2) or perform a rule shift (e.g. Texture 1 to Odor 1; Fig 3A and 3B). There were no differences between experimental groups in learning to associate either odor or digging media to the food reward during the initial association (Fig 3C). During the OFC-dependent reversal task, TBI animals committed significantly more errors before they successfully unlearned the initial rule and associated the new stimulus with the food reward (Fig 3D; p<0.05). Conversely, there was no effect of TBI on the animals’ ability to learn a new initial association the following day (data not shown) or perform the mPFC-dependent rule shift (Fig 3E).

Furthermore, because TBI-induced symptoms in human patients persist years after injury [3, 4], we examined a separate cohort of mice at 5.5 months after TBI surgery to determine whether OFC function would be chronically impaired. Similar to the first group of TBI animals, the second TBI cohort was also impaired on OFC-dependent reversal and unaffected on initial association and mPFC-dependent rule shifting (Fig 3C and 3D; p<0.05).

### Anxiety and hippocampal-dependent memory are unaffected by frontal TBI

**Fig 4:**
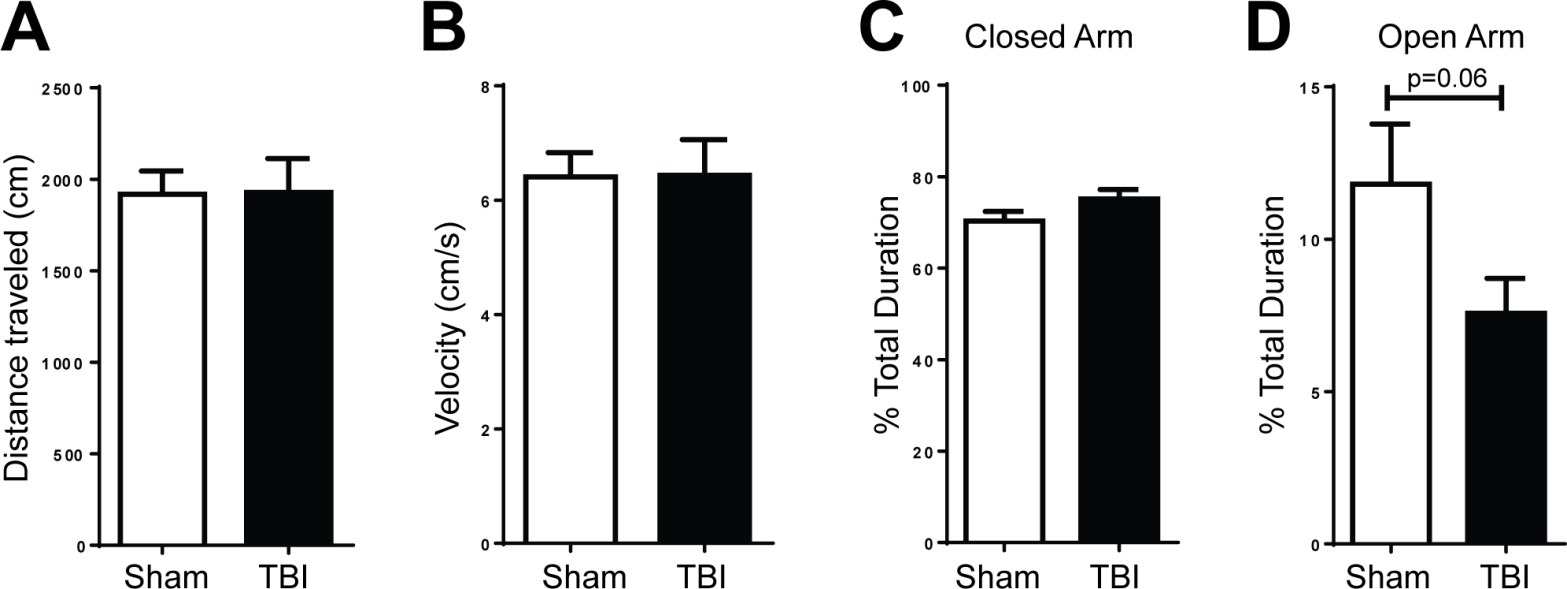
Animals with frontal lobe TBIs exhibit a trend for increased anxiety on the elevated plus maze at 1 month post-injury. **A.** Injured animals traversed as much distance as sham animals did (Student’s t-Test; p>0.05) and **B.** displayed no difference in movement speed (Student’s t-Test; p > 0.05). **C.** Injured animals did not spend more time in the closed arms (Student’s t-Test; p = 0.11), but **D.** did have a trend for less time spent exploring the open arms of the maze (n = 21-22/group; Student’s t-Test; p = 0.06).

To determine whether a frontal TBI would affect anxiety, we tested animals a month after injury on the EPM which measures anxiety as the time animals spend in the closed and open arms of the maze. Frontal contusion did not reduce the distance traveled or affect the average velocity of the animal compared to sham surgery controls, indicating no loss of motivation to explore or impairment in motor control (Fig 4A and 4B). There was no significant difference on the time spent in the closed arms of the EPM between sham and injured mice (Fig 4C; p = 0.112). We did observe a trend for TBI animals to spend less time in the open arm of the EPM compared to sham although the result was not statistically significant (Fig 4D; p = 0.065).

**Fig 5:**
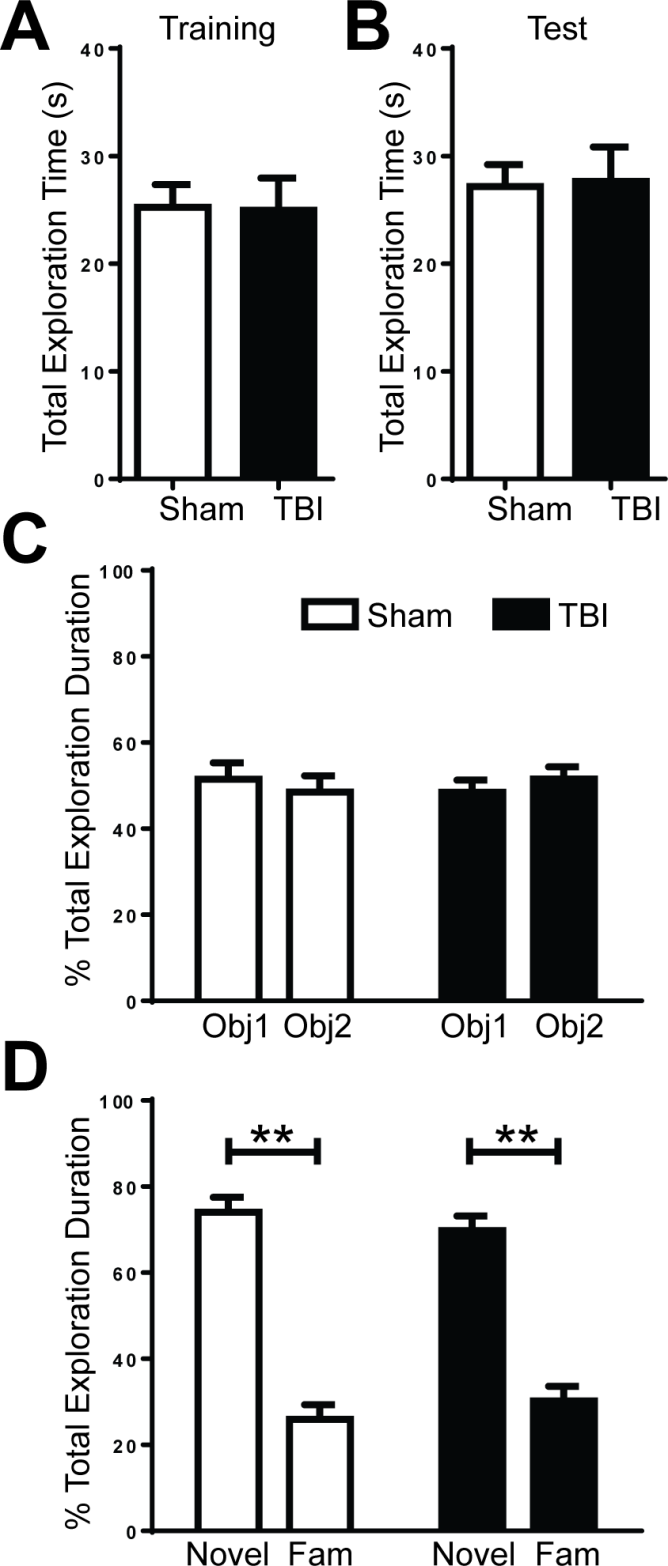
Frontal lobe TBI does not affect recognition memory on hippocampal-dependent novel object recognition task. **A, B.** Sham and TBI animals did not differ significantly on their exploration time during training (A) or test (B) trials (Student’s t-Test; p > 0.05). **C.** There was no preference for either of the two identical objects (Obj1, Obj2) in sham (white) and TBI (black) animals (Student’s t-Test; p > 0.05). **D.** Animals were tested 24 hours after the initial training and both sham (white) and TBI (black) animals significantly preferred to explore the novel object (Novel) over the familiar object (Fam) (n = 9/group; Student’s t-Test; **p < 0.01).

Additionally, to assess whether frontal lobe TBI would affect hippocampal-dependent memory, the animals were tested on the hippocampal-dependent novel object recognition assay. During the training trial, animals were exposed to two identical objects. On the test trial 24 hours after, animals were presented with one familiar object from the training trial and a novel object. Sham and TBI animals explored for the same amount of time during both training and testing trials (Fig 5A and 5B). Animals from both groups explored the identical objects equally during the training trial (Fig 5C). Furthermore, both sham and TBI animals exhibited similarly significant preference for the novel object over the familiar object during the test trial, suggesting hippocampal function was unaffected by the injury (Fig 5D; p<0.01).

## Discussion

Currently, there is a lack of analogous, behaviorally characterized TBI models investigating frontal lobe injuries despite the prevalence of focal contusions to the frontal lobe in TBI patients [5, 25, 26]. Utilizing the CCI model to simulate a frontal contusion, we evaluated three frontal lobe dependent behaviors to determine whether TBI symptoms observed in human patients could be recapitulated in mice. Given the functional and anatomical connection between PFC and hippocampus [20], we also assessed performance on a hippocampal-dependent assay.

Social behavior is commonly affected among human patients leading to impairment of social cognition such as emotion recognition, theory of mind, and empathy [24, 27]. Furthermore, the degree of social behavioral deficits has been correlated to severity of injury based on duration of post-TBI amnesia and formation of lesion [14]. Recent studies have shown that blast TBI and pediatric TBI rodent models demonstrate impairments in sociability and social recognition, recapitulating changes in social behavior seen in human patients with analogous injuries [16, 28]. While our frontal contusion model exhibits normal sociability, the injured mice have a decreased preference for social novelty. The result warrants further investigation as the impairment in social recognition could be due to social anxiety, diminished response to novelty, or deficits in forming social memory [22, 29, 30]. Our data from the EPM and the novel object recognition assay, however, suggests that any effect of TBI to anxiety or preference for novelty may be specific to social interactions.

In addition to social behavior, the frontal lobe is also responsible for maintenance of attention and the ability to reverse recently acquired behavioral rules [31, 32]. TBI patients often exhibit deficits in cognitive flexibility as reflected by poor performance on the Wisconsin Card Sorting Test in which participants learn initial rules for matching cards into sets and then have to unlearn previous associations in subsequent trials that follow new rules for organization [33, 34]. This diagnostic test has been adopted for rodents in the form of the rule shift and attentional set shift assays, allowing for quantification of an animal’s ability to reverse out of a previously learned behavioral rule and its ability to switch attention from one stimulus to another [17, 31, 35]. A previous study found that a severe parietal CCI injury that resulted in substantial loss of brain tissue could induce deficits in both reversal and set shifting ability three weeks post-injury [36]. Our frontal lobe contusion model, however, produced moderate cortical tissue loss and exhibited deficits only on rule reversal and not on rule shift behavior which suggests that reversal ability may be more vulnerable to frontal lobe TBIs in mice. Furthermore, the impairment in reversal learning persisted past 5 months after injury, suggesting the effect of injury is chronic as it is in human patients.

Patients also often develop chronic anxiety and comorbid anxiety-related disorders after TBI events [12, 37, 38]. Various models of TBI on the parietal lobe however have produced conflicting effects of injury in rodents; some models report increased anxiety while others exhibit reduced anxiety or no significant changes in behavior [39–41]. Further complicating the matter, our current data shows a trend for anxiogenic effect of injury which may suggest this injury model is insufficient to induce significant anxiety-like behavior. This is supported by data in human survivors of TBI showing that severity of injury is more highly correlated with anxiety disorders [42].

The behavioral sequelae observed in this study suggest an OFC-specific vulnerability to frontal TBI. This is primarily supported by the deficit on reversal learning observed in the injured mice. Lesion studies and optogenetic manipulations on mice performing the rule shift assay have shown that the OFC is necessary for reversal learning while rule shifting depends on mPFC function [17, 31]. The region-specificity of the behavioral assay is further supported by models for schizophrenia and aging that display deficits in OFC neuronal function and reversal learning only [31] or mPFC function and rule shifting only [43, 44]. In humans, the OFC is commonly injured after severe trauma and the TBI patients often display behavioral symptoms, such as poor performance on the Wisconsin Card Sorting Test, similar to those with explicitly degenerated or lesioned OFCs [5, 10, 33]. Moreover, OFC involvement has also been documented in social recognition impairment and development of anxiety in patients with anxiety disorders [45, 46].

The specificity of impairment to the OFC further distinguishes the frontal CCI model from parietal CCI models. Numerous studies on the network between the hippocampus and frontal cortex have established that the ventral hippocampus (posterior parietal cortex; PPC) projects to the frontal cortex and vice versa [47]. The necessity of the hippocampus in reversal learning and rule shifting has been highlighted in two studies via chemical lesioning of the ventral hippocampus or PPC [48, 49]. Taken with the fact that the CCI lesion in the Bondi et al 2014 paper is an extremely severe injury that essentially ablates the hippocampus in one hemisphere, it follows that the parietal CCI can affect both reversal and set shift. In contrast, our frontal CCI model does not lesion either the OFC or mPFC, and hippocampal function is unaffected as measured by the novel object recognition assay. Furthermore, since mPFC-dependent rule shifting is unimpaired, our data implies that the frontal CCI only affects OFC-involved pathways. It is also possible that OFC-specific function is particularly vulnerable to frontal CCI independent of projections to the region. Investigating the OFC further may allow us to identify cell populations and the underlying mechanisms affected by injury that lead to development of behavioral dysfunction.

The frontal CCI mouse model provides a behavioral foundation for exploring the effect of TBI on frontal lobe regions and their functionalities. The model recapitulates OFC-dependent deficits seen in human patients while sparing the function of the mPFC and hippocampus [11, 50, 51]. The specificity of the model’s behavioral outcome distinguishes the OFC as a region of interest for future studies to differentiate the effects of parietal and frontal contusions and identify potential mechanisms that may not be affected in parietal injury models without severe damage to the brain. Further investigation of the physiological effects of frontal TBI in the OFC could help elucidate particularly vulnerable cell type populations and molecular targets to improve treatment for human TBI patients.
